# ISU *FLU*ture: a veterinary diagnostic laboratory web-based platform to monitor the temporal genetic patterns of Influenza A virus in swine

**DOI:** 10.1186/s12859-018-2408-7

**Published:** 2018-11-01

**Authors:** Michael A Zeller, Tavis K Anderson, Rasna W Walia, Amy L Vincent, Phillip C Gauger

**Affiliations:** 1 Bioinformatics and Computational Biology Program, Iowa State University, Ames, IA USA; 2 Department of Veterinary Microbiology & Preventive Medicine, Iowa State University, Ames, IA USA; 3 Virus and Prion Research Unit, National Animal Disease Center, USDA-ARS, Ames, IA USA; 4 Department of Veterinary Diagnostic & Production Animal Medicine, Iowa State University, 1575 Vet Med, 1850 Christensen Dr, Ames, IA 50011-1134 USA

**Keywords:** Influenza a virus, Epidemiology, Swine, Zoonotic diseases, Vaccines, Virus evolution, H1N1, H1N2, H3N2

## Abstract

**Background:**

Influenza A Virus (IAV) causes respiratory disease in swine and is a zoonotic pathogen. Uncontrolled IAV in swine herds not only affects animal health, it also impacts production through increased costs associated with treatment and prevention efforts. The Iowa State University Veterinary Diagnostic Laboratory (ISU VDL) diagnoses influenza respiratory disease in swine and provides epidemiological analyses on samples submitted by veterinarians.

**Description:**

To assess the incidence of IAV in swine and inform stakeholders, the ISU *FLU*ture website was developed as an interactive visualization tool that allows the exploration of the ISU VDL swine IAV aggregate data in the clinical diagnostic database. The information associated with diagnostic cases has varying levels of completeness and is anonymous, but minimally contains: sample collection date, specimen type, and IAV subtype. Many IAV positive samples are sequenced, and in these cases, the hemagglutinin (HA) sequence and genetic classification are completed. These data are collected and presented on ISU *FLU*ture in near real-time, and more than 6,000 IAV positive diagnostic cases and their epidemiological and evolutionary information since 2003 are presented to date. The database and web interface provides rapid and unique insight into the trends of IAV derived from both large- and small-scale swine farms across the United States of America.

**Conclusion:**

ISU *FLU*ture provides a suite of web-based tools to allow stakeholders to search for trends and correlations in IAV case metadata in swine from the ISU VDL. Since the database infrastructure is updated in near real-time and is integrated within a high-volume veterinary diagnostic laboratory, earlier detection is now possible for emerging IAV in swine that subsequently cause vaccination and control challenges. The access to real-time swine IAV data provides a link with the national USDA swine IAV surveillance system and allows veterinarians to make objective decisions regarding the management and control of IAV in swine. The website is publicly accessible at http://influenza.cvm.iastate.edu.

**Electronic supplementary material:**

The online version of this article (10.1186/s12859-018-2408-7) contains supplementary material, which is available to authorized users.

## Background

Influenza A virus (IAV) causes respiratory disease in swine that decreases health and wellbeing through morbidity and mortality, and impacts production through increased costs associated with vaccination, treatment, and increased biosecurity programs [1]. It has been estimated that IAV costs the pork industry as much as $1 billion annually due to animal health care expense and increased production times [1, 2]. Two consistent contributors to failures of control efforts are based on the ecology of swine IAV: continual evolution of endemic IAV lineages and infection by IAVs from different species. The introduction of human IAV to swine populations occurs relatively frequently [3–7], and has resulted in four major lineages that currently circulate in US swine. Consequently, there is a large diversity of co-circulating genetic and antigenic lineages of IAV within the three predominant subtypes, H1N1, H1N2, and H3N2 [8].

The four major lineages of swine IAV in the US may be further divided based on their genetic diversity and antigenic phenotype. The H1 classical swine lineage emerged coincident with the 1918 Spanish flu in humans [9] and diversified to contain five distinct clades of viruses: H1-α (global nomenclature 1A.1 and 1A.1.1); H1-β (1A.2); H1-γ2 (1A.3.2); H1-pdm09 (1A.3.3.2); and the H1-γ (1A.3.3.3) [10–14]. A second H1 lineage was detected in the 2000s [13], the result of two separate human-to-swine spillovers: these include clades H1- δ1 (1B.2.2, 1B.2.2.1, 1B.2.2.2) and H1- δ2 (1B.2.1). The H3 lineages in swine also reflect multiple human-to-swine spillovers, and were the result of two independent introductions more than 10 years apart. The first H3 lineage in United States (US) swine emerged in 1998, and was derived from a triple reassortant virus composed of genes derived from human, avian, and swine origin, denoted as Cluster IV [4, 15]. Over 20 years, this lineage diversified into six genetically different clades that were designated Cluster IVA through F [16]. The second major H3 introduction was first detected in swine in 2012, and was most closely related to the human seasonal H3 IAV from the 2010–2011 human influenza season [17]. Additionally, the neuraminidase (NA) genes of the N2 subtype are derived from the 1998 H3N2 introduction (colloquially named “1998” N2) or the 2000s-human seasonal H1 introduction (colloquially named “2002” N2). The N1 subtype may be classified as a derivative from the 1918 H1N1 introduction (classical swine N1) or from the 2009 H1N1 pandemic (pandemic N1). A further consequence of this observed diversity, and the segmented nature of the influenza genome, is the process of reassortment that may occur during coinfections in swine [12, 14]. These reassortment events and adaptive mutations to swine hosts create novel viruses that have the potential to be reintroduced into the human population, posing a serious human health risk [18]. Consequently, interventions with effective vaccines that reduce replication, transmission, and pathology of swine IAV benefit the health and wellbeing of pigs, economic position of swine producers, and benefit public health through reduction of a potential zoonotic pathogen [19, 20].

The current standard for controlling IAV are multivalent vaccines focused on immunity against the major surface glycoproteins, hemagglutinin (HA) and NA [21]. A vaccine that contains strains with relatively high genetic relatedness to circulating strains is generally expected to have potential for higher efficacy [22–24]. Prior work has shown that traditional inactivated vaccines can quickly lose cross-reactivity to antigenically drifted heterologous IAV [23]. Consequently, an approach to improve vaccine efficacy is to match the strains used in multivalent vaccines with the diversity of viruses circulating in the field [25, 26]. This process requires regular surveillance, analysis, and correlates of protection linked to changes in the genetic diversity of co-circulating swine IAV subtypes. From 2009 to present, the United States Department of Agriculture (USDA) voluntary swine IAV surveillance system has made great improvements in the availability of sequence data from swine IAV [8, 26]. However, these data are derived from voluntary participation by producers and veterinarians and may not be representative of all IAV in all states or regions. Additionally, the lag in time between a diagnosis to the release of IAV sequences in public databases may impact the ability of vaccine manufacturers to rapidly update vaccines or for veterinarians and producers to modify intervention efforts.

To address this gap in timely information on IAV in swine, ISU *FLU*ture was designed for near real-time visualization of trends in genetic diversity and how IAV is changing spatially and temporally, with sequences derived from respiratory samples submitted to the Iowa State University Veterinary Diagnostic Laboratory (ISU VDL). ISU *FLU*ture provides an interactive environment where epidemiological data linked to sequences can also be evaluated at near real-time. A unique strength of this platform is its integration into previously privately held diagnostic test result data from the largest swine diagnostic laboratory in the US; consequently, the data on ISU *FLU*ture is the most comprehensive reflection currently available of the activity of IAV in swine in the US. A suite of tools has been designed to analyze trends in the metadata and sequence data. The ISU *FLU*ture platform complements other resources for IAV in swine such as the Influenza Research Database [27, 28] by providing access to veterinary diagnostic results and metadata that may not be submitted to public databases due to client privacy or programmatic restrictions as well as a real-time perspective on swine IAV epidemiology and evolution.

## Construction and content

### The Iowa State University Veterinary Diagnostic Laboratory

The ISU VDL provided IAV diagnostic and sequencing services from 2003 to present, with significant increases in the past 2 years: since 2015, the ISU VDL processed over 1,000 IAV-related cases annually collected from 38 states across the continental US. The IAV diagnostic results and related sequences are stored in the ISU VDL Laboratory Information Management System (LIMS), a private database of client records. Of the IAV positive diagnostic submissions, approximately 49% of IAV cases were eligible for the USDA IAV swine surveillance program beginning in 2009 [8], and for these submissions, the sequence data is publicly released to NCBI GenBank [29]. Consequently, the ISU VDL has accumulated over thirteen years of IAV diagnostic and sequence data in swine, of which a majority is not currently accessible by the public or stakeholders. These data include pig specific information such as age and location, as well as HA and NA nucleotide sequences. This information represents a unique and valuable resource that enables the determination of IAV evolutionary trends and spatial and temporal dynamics that have previously been inaccessible.

### Data collection

Veterinarians submit diagnostic samples (lung tissue, nasal swabs, or oral fluids) collected on farms from swine with clinical signs (e.g., coughing, dyspnea, fever, depression, lethargy) for IAV screening using real-time reverse transcription polymerase chain reaction (RT-qPCR). Information related to age, weight, and farm location may also be provided at the discretion of the submitter. Samples that are RT-qPCR positive for IAV are subtyped to determine the HA and NA. Veterinarians may then elect to participate in the USDA IAV swine surveillance program, which subsidizes HA and NA sequencing and virus isolation if the case qualifies based on RT-qPCR cycle threshold (CT) values of ≤25 for lung and nasal swab samples and ≤ 20 for oral fluid samples. Samples that meet these criteria receive a unique USDA barcode (a nine-digit alpha-numeric designation beginning with A0) and the resulting sequences are publicly available in NCBI GenBank while maintaining ISU VDL client confidentiality. The client may elect to pay for private diagnostic services within the ISU VDL system with or without participation in the USDA system. If a veterinarian or producer does not want to participate in the USDA IAV surveillance program, or if the diagnostic sample does not meet USDA requirements for inclusion, they may choose to pay for sequencing using the ISU-VDL criteria of a screening RT-qPCR of CT ≤ 38. If successful, a sequence for the HA and NA may be acquired. Cases may also be submitted anonymously in the USDA program if initial CT values qualify and at the discretion of the ISU VDL. Through the anonymous USDA submission, all ISU VDL client information is removed and sequence and isolates are submitted with only state-level information. However, in the ISU VDL LIMS, the sequence data is linked with client-provided information regarding age, weight, and farm location and additional diagnostic information collected from the sample such as influenza subtyping PCR results and other pathogen identification in cases of respiratory disease with multi-etiologic diagnoses.

### Data curation

The swine IAV cases from LIMS were extracted and curated in an independent SQL database for ISU *FLU*ture to allow additional processing of the data to prepare it for display on the ISU *FLU*ture webpage. Diagnostic cases maintained privately at the ISU VDL were non-redundantly combined with ISU VDL cases submitted as part of the USDA swine IAV surveillance program using a unique identifier and curated in the ISU *FLU*ture Database. Updates to the ISU *FLU*ture database occur at daily intervals. The data were reduced to USDA accession ID (where applicable), received date, data source (USDA or ISU VDL diagnostic streams), specimen used for PCR detection, specimen used for sequencing, pig age in days, pig weight in pounds, the geographic location (at US state resolution), the IAV subtypes detected in the specimen, the HA sequence, and the NA sequence (for cases included in the USDA IAV swine surveillance system). The case associated information was voluntarily provided by the clients, thus not all variables were available for every case. Duplicate cases were removed from the results in instances where multiple diagnostic samples were submitted from the same farm, retaining only the sample that contained the HA sequence, or the sample that tested positive for IAV when sequencing failed, but a subtype was available.

The description of the evolutionary dynamics of the sequenced samples was achieved by inferring the HA and NA phylogenetic clade for each case where applicable. HA clades are initially screened using a logistic regression one-vs-all multiclass classifier trained with all cases currently in the database with known clades to flag sequence data that would need follow up. HA clades for H1 subtype were determined using the *Swine H1 Clade Classification Tool* available on the Influenza Research Database [26, 27, 30]. The results were reported in the US familiar clade terms as the primary stakeholders for the ISU *FLU*ture website, US veterinarians and producers, would not be versed in the global H1 nomenclature and the global context would not frequently be relevant for the US-restricted data. The H3, N1, and N2 clades were determined by phylogenetic analysis from a set of reference sequences (Additional file 1: Figure S1 and Additional file 2; Additional file 3: Figure S2 and Additional file 4; Additional file 5: Figure S3 and Additional file 6). Nucleic acid sequences for each case in question were included with the reference sequences and aligned with MAFFT v7.271 [31] using default settings. FastTree2 v2.1.9 [32] was used to infer the best-known maximum-likelihood tree for each of the gene alignments implementing a general time reversible model of nucleotide substitution with a CAT model of rate heterogeneity with branch lengths rescaled to optimize the Gamma20 likelihood [32]. The HA and NA phylogenetic clade for each strain was subsequently assigned [10–14, 16, 17, 33]. The ISU VDL reports any novel IAV to the USDA as per the influenza surveillance guidelines in swine. Unique influenza viruses detected in swine may be reported to the World Organization for Animal Health (OIE) at the discretion of the USDA. USDA is responsible for diagnosing and reporting of OIE listed IAV. Resultant data was checked for irregularities such as mismatched clades and subtypes before being inserted into the underlying ISU *FLU*ture database. The relational database stores information related to the USDA or ISU VDL case accession number, sample receipt date, data source, animal age, animal weight, animal location, sample used for PCR subtyping, subtyping PCR results, the specimen used for sequencing, the HA clade, and NA clade; and was internally identified by auto-generated case names based on this information.

### Determining trends in IAV with interactive visualization tools

We developed multiple interactive tools within ISU *FLU*ture to visualize IAV dynamics using the JavaScript libraries C3, D3, jQuery, and Raphael [34, 35]. Currently, four different modes of interpretation exist; correlation, time series, regional, and heat-map. The correlation tool depicts the relationship between two different database variables, facilitated using C3’s bar graph functionality. The X-axis displays the unique or binned values of a single variable for which the axis displays the count of occurrences in the database. Data normalization is built into the system, where the different variables in a bin are summed and divided by the total. The time series tool uses C3’s time series chart functionality to display the binned counts of data over time with the granularity of day, week, month or year. The heat-map tool displays HA and NA clade pairings which are displayed in a table over a selected region of time for cases where both the neuraminidase and the hemagglutinin phylogenetic clades are available. Monochromatic coloration is applied to the table to emphasize higher representation by intensity. The regional tool is designed to show the geographic provenance of the data in ISU *FLU*ture. A map of the United States is drawn using the Raphael library, with the different states shaded proportional to the number of swine cases in the database over a selected range of time. The number of cases are reported numerically in a table under the map.

## Utility and discussion

**Fig. 1:**
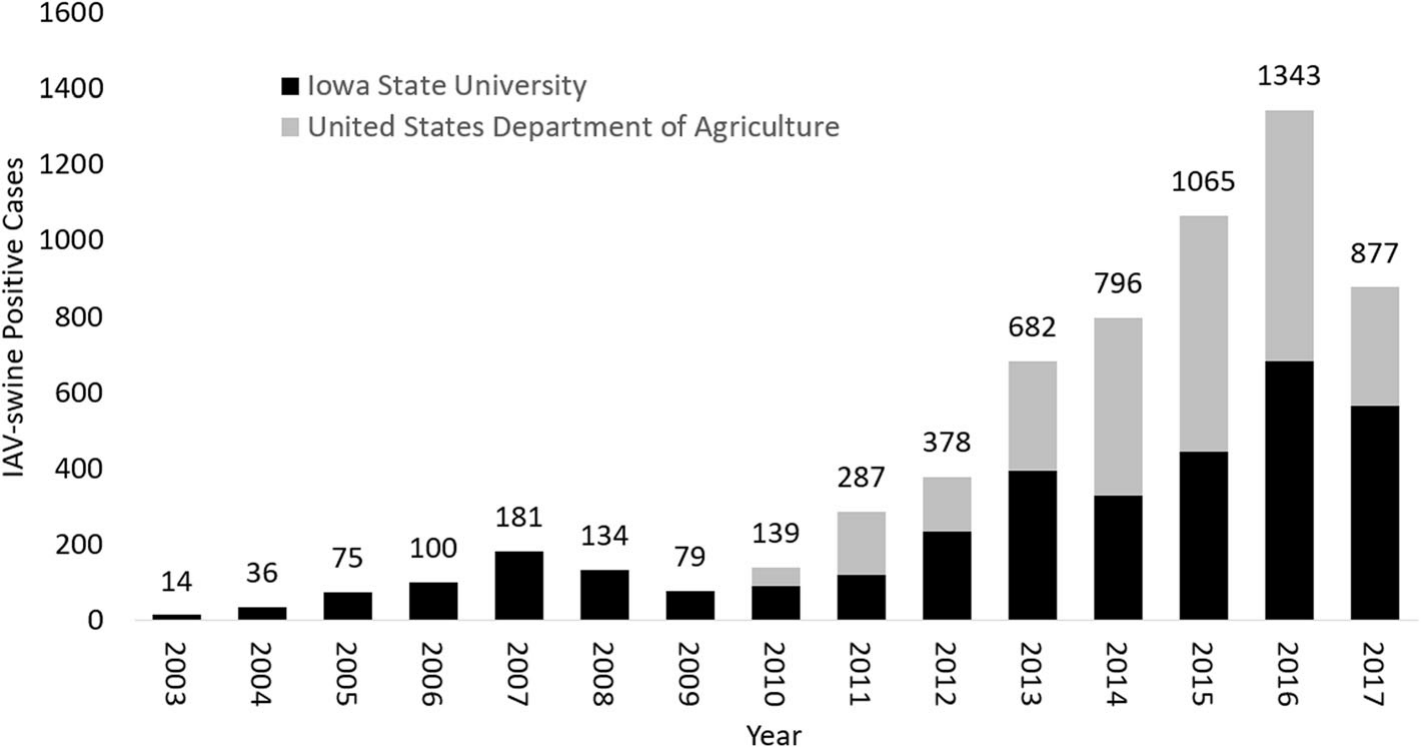
The number of influenza A virus (IAV) positive swine cases visualized by ISU *FLU*ture, separated by year of submission. Diagnostic cases maintained privately at the Iowa State University Veterinary Diagnostic Lab (ISU VDL) were non-redundantly combined with ISU VDL cases submitted as part of the United States Department of Agriculture (USDA) swine IAV surveillance program and curated in the ISU *FLU*ture Database. USDA cases are in gray, and the cases exclusive to the ISU VDL and ISU *FLU*ture are in black. Sequences generated from cases submitted as part of the USDA surveillance program are accessible to the public through GenBank

The ISU *FLU*ture database and interactive website provides a unique web resource that offers thirteen years of case summaries for IAV in swine. The database currently has 6,186 unique cases (Fig. 1), 5,405 HA clade designations, and 2,206 NA clade designations. Of all cases, less than half (2,991) were submitted to the USDA IAV swine surveillance program with sequences publicly accessible through NCBI GenBank. Future updates to ISU *FLU*ture plan to include phylogenetic analysis and interfacing with other public IAV databases such as the Influenza Research Database and the Influenza Virus Resource [29, 30].

The primary utility of the ISU *FLU*ture database is near real-time access to the number of IAV detections and variation in HA and NA clades over time. Both publicly and privately funded case data is available within days after all appropriate diagnostic tests are complete, dramatically reducing the time traditionally needed for a diagnostic case to be sequenced and then shared in a public venue. ISU *FLU*ture is not designed to compete with other public sequence databases such as NCBI GenBank or with web-based analytic tools provided by the Influenza Research Database, but rather to work in conjunction with them. Information can be more rapidly disseminated to ISU *FLU*ture, as additional action is required to submit and release sequence data to these public databases. Furthermore, ISU *FLU*ture is designed as a graphical interactive analytical tool specific for IAV detected in US swine populations, aiming to find trends in swine IAV case data based upon parameters determined by the user.

### Selecting vaccine strains to improve protection

ISU *FLU*ture was created to assist stakeholders, namely veterinarians, producers, vaccine developers, and other researchers, in making informed decisions about controlling IAV through matching the components of vaccines with currently circulating IAV strains of similar diversity. To achieve this, ISU *FLU*ture displays the trends in the genetic diversity of the major surface glycoproteins, HA and NA, of swine IAV. Some HA genetic clades may be restricted to specific regions and/or to a contemporary temporal window and the HA of vaccine strains need to be periodically updated or customized to specific herds. Additionally, ISU *FLU*ture visualizes the commonly paired NA with each of the major HA clades under the heat map tool. Studies have shown that vaccines targeting the NA provide protection [36] and vaccines that match the HA and NA with the challenge virus are more effective, reduce the chance of vaccine failure, and reduce the likelihood of vaccine associated enhanced respiratory disease [36, 37]. Consequently, ISU *FLU*ture enables identification of dominant HA/NA pairings of contemporaneously circulating strains.

**Fig. 2:**
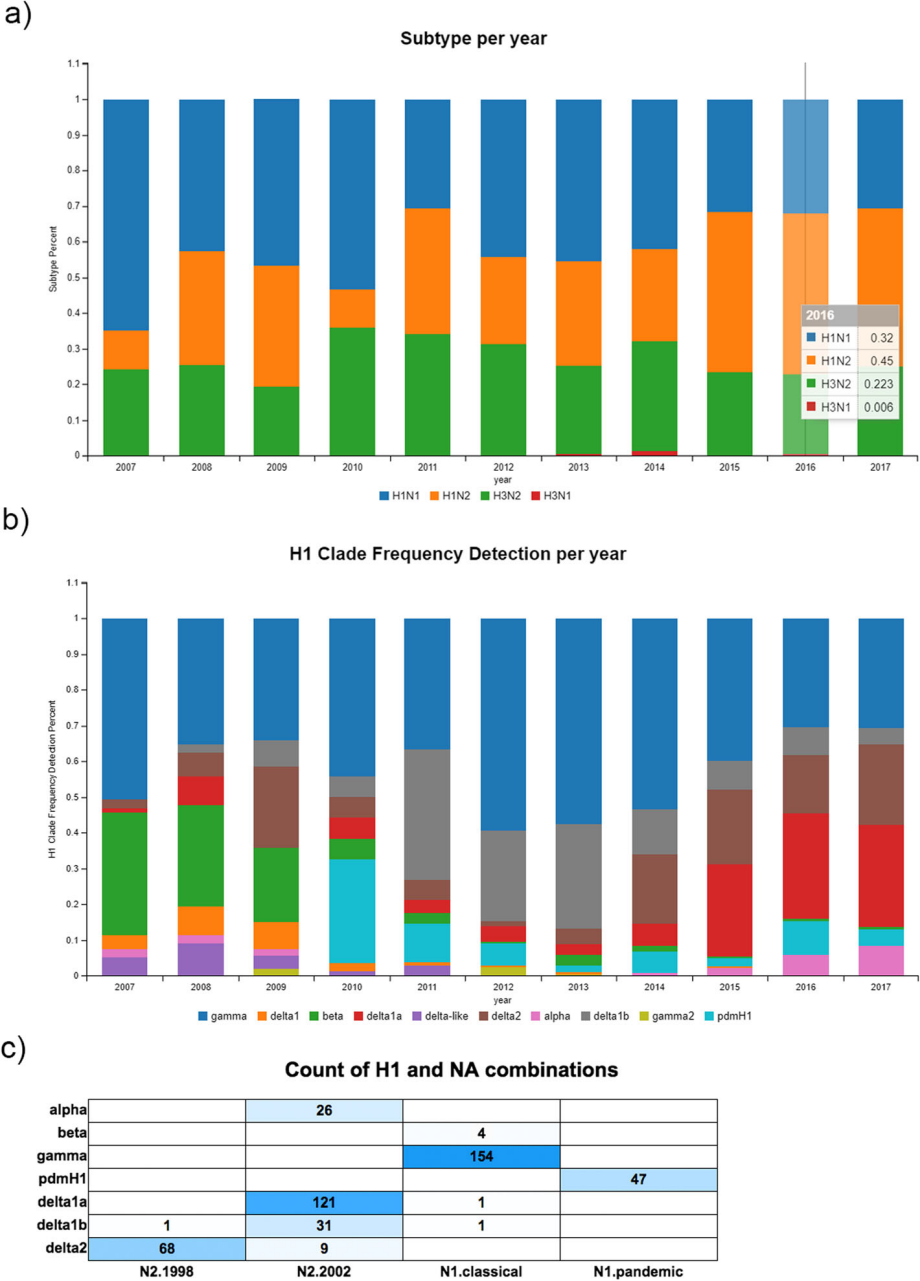
Relative frequency of subtype, H1 clades, and HA and NA pairing. (**a**) The percent of subtypes of influenza A virus (IAV) positive cases processed at the Iowa State University Veterinary Diagnostic Laboratory (ISU VDL) annually. H1N1 is presented in blue, H1N2 in orange, H3N2 in green, and H3N1 in red. (**b**) The percent of H1 clades detected per year at the Iowa State University Veterinary Diagnostic Laboratory (ISU VDL). The colors represent the following: H1-α: pink (1A.1 and 1A.1.1), H1- β (1A.2): green, H1- γ (1A.3.3.3): blue, H1- γ2 (1A.3): olive, H1- δ1 (1B.2.2): orange, H1- δ1a (1B.2.2.1): red, H1- δ1b (1B.2.2.2): gray, H1- δ2 (1B.2.1): brown, H1- δ-like (1B.2.2): purple. (**c**) The combination of hemagglutinin clade and neuraminidase clade pairings in H1 Influenza A virus (IAV) isolates sequenced at the Iowa State University Veterinary Diagnostic Laboratory (ISU VDL) for H1 viruses sequenced between January 1, 2016 to January 1, 2017

The following outlines a general example of how to use the ISU *FLU*ture database to identify potential vaccine strains by identifying the dominant HA and NA clades. Plotting the year against the subtype in the correlation tool shows that H1 viruses accounted for 77% of the observed IAV infections in swine during 2016 (Fig. 2a), indicating a need for improved H1 vaccines. Further, the H1 clade correlation tool revealed that the primary HA phylogenetic clades were H1-γ (1A.3.3.3), H1- δ1a (1B.2.2.1), and H1- δ2 (1B.2.1) at 30%, 29%, and 16% respectively (Fig. 2b). Additionally, the composition of the HA clade and NA clade pairs observed within H1 subtype viruses were visualized using the Heat Map tool, with the top three NA clades represented independent of HA being the N2.2002, N1.classical, and the N2.1998. The top three HA-NA pairings observed from the heat map are the H1-γ (1A.3.3.3) paired with N1.classical represented in 33.2% of the cases, H1- δ1a (1B.2.2.1) paired with N2.2002 represented in 26.1% of the cases, and H1- δ2 (1B.2.1) paired with N2.1998 represented in 14.6% of cases (Fig. 2c). Using this knowledge, H1 vaccines are minimally needed to target H1-γ (1A.3.3.3), H1- δ1a (1B.2.2.1), H1- δ2 (1B.2.1), N1.classical, N2.2002, and N2.1998. The N2.2002 was also present in about 97% of detected H3s in 2016, adding further emphasis on the importance of this component. From January 2017 to September 2017 the pattern of H1 detections shifted. The H1 clade correlation tool showed that the primary HA phylogenetic clades were H1-γ (1A.3.3.3), H1- δ1a (1B.2.2.1), and H1- δ2 (1B.2.1) at 30%, 28%, and 22%. The increase in the H1- δ2 (1B.2.1) came at the cost of other less represented clades.

### Identification of emerging viruses and reassortment events

**Fig. 3:**
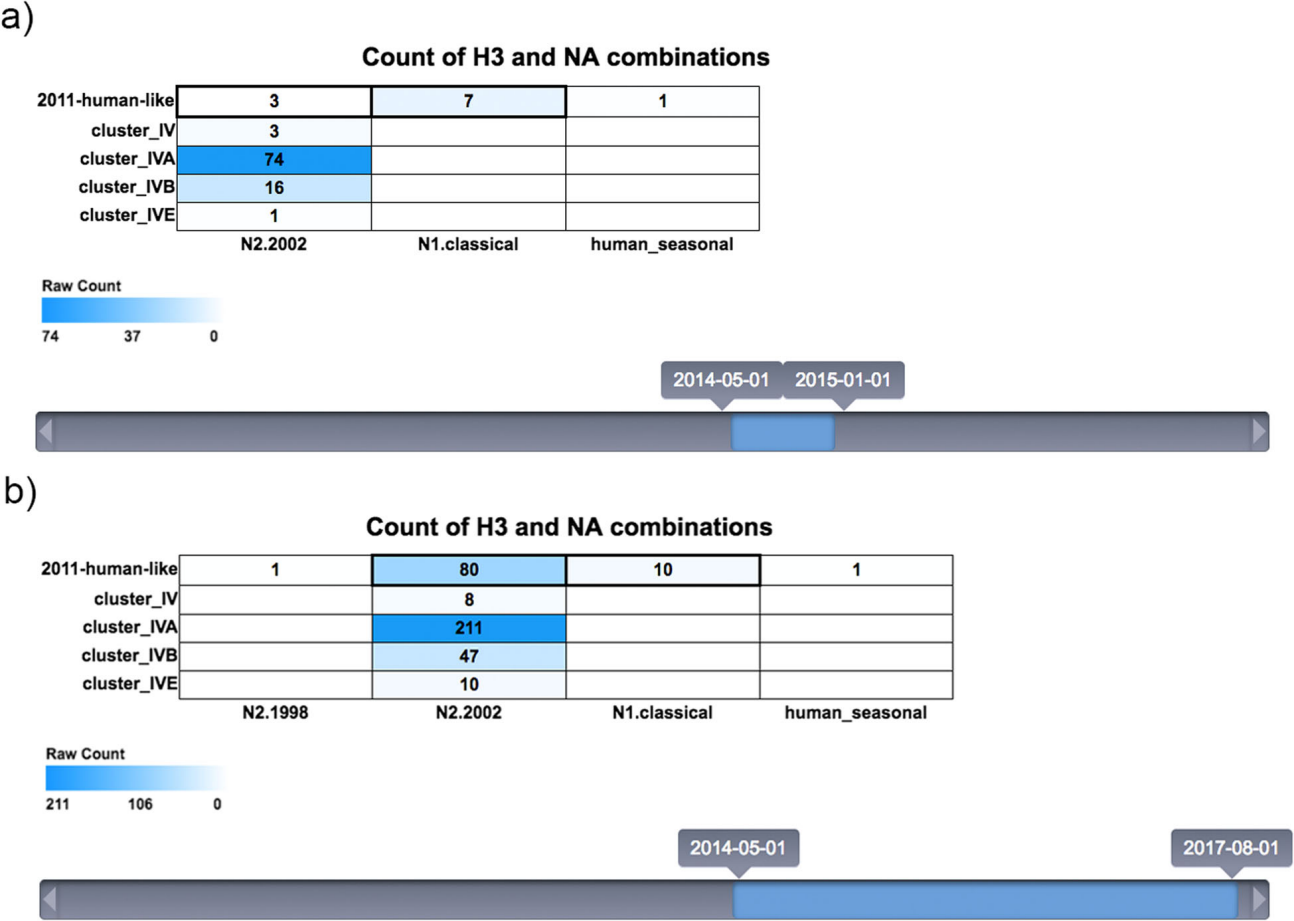
The combination of hemagglutinin clade (y-axis) and neuraminidase clade (x-axis) pairings in influenza A virus (IAV) isolates sequenced at the Iowa State University Veterinary Diagnostic Laboratory (ISU VDL). (**a**) From May 1 of 2014 and January 1 of 2015, the human-like HA in swine was primarily paired with the N1 classical clade. **(b**) After January 1 of 2015, there was a switch to a predominant pairing of the human-like H3 with the N2.2002 NA and a subsequent proliferation of the human like HA

**Fig. 4:**
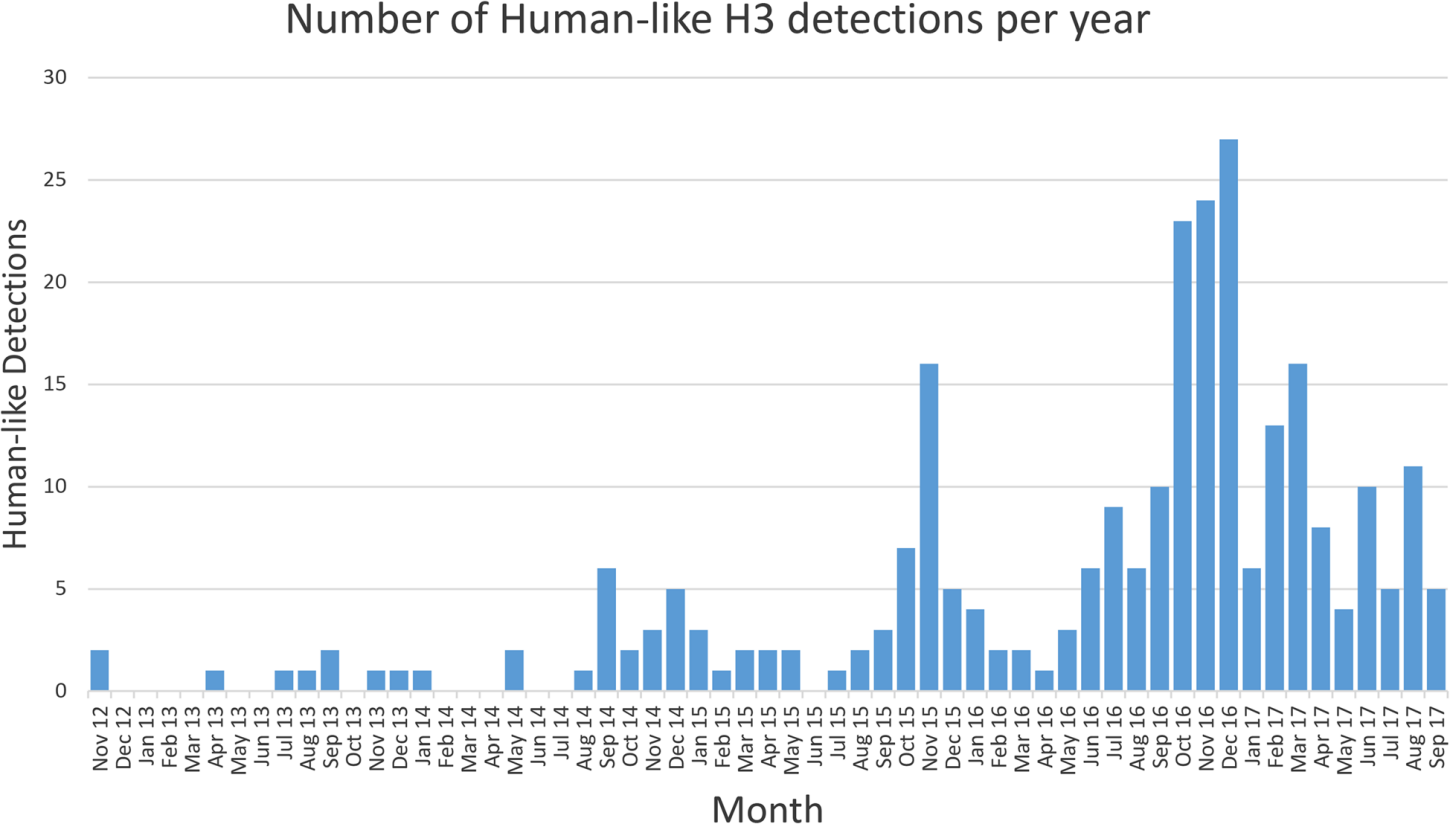
The number of human-like H3 clade IAV detected in swine per month at the ISU VDL from October of 2012 to July of 2017. Since the initial detection in October 2012, the strain has established itself endemically in swine, with seasonal peaks matching the onset of the flu season

The introduction of novel lineages following interspecies transmission episodes [38], as well as genetic mutation and reassortment, result in evolution of swine IAV and antigenic shift and drift [15, 39]. An intelligent early-warning system [40] that detects emergence of novel influenza virus lineages in swine could facilitate control prior to these new lineages becoming widespread. For example, the ISU *FLU*ture web portal was utilized to detect and track the evolution of a novel H3 lineage in swine. In 2012, a human-origin H3N2 virus was detected at the ISU VDL [17] and the first NA sequence associated with the human-like H3 clade was detected in May 2014 as a human-like N2 (Fig. 3a). Then, from May 2014 to December 2014, the primary NA for the human-like H3 was the N1.classical swine subtype. This was of note since the H3N1 subtype of any genetic lineages has failed to demonstrate sustained transmission in pigs over prolonged periods of time. In November 2014, a human-like H3 with the N2 belonging to the swine N2.2002 phylogenetic clade was first detected, and by April 2015, the human-like H3 HA had become primarily associated with the N2.2002 NA while the N1.classical ceased to be detected with the human-like H3 (Fig. 3b). These viruses were antigenically characterized [17], demonstrating that current swine vaccines containing the previously dominant Cluster IV H3 were unlikely to provide adequate protection and should be updated. Prior to January 2015 there were 32 detections of human-like H3. After January 2015 through September 2017, 236 new detections were noted (Fig. 4), consistent with the spread of a novel virus in a naïve population. Additionally, ISU *FLU*ture can be used to visualize the spatial dissemination of this lineage from 2 states in 2014, to 12 states in 2017 (data not shown).

While the human-like H3 is of utmost importance to the swine industry, this lineage of viruses was also recently detected as zoonotic events in humans that attended agricultural fairs who had contact with swine in 2016 [41] and 2017 [42, 43], designated as a variant H3N2 (H3N2v) in humans. This demonstrates how the data from ISU *FLU*ture can be used to identify emerging novel variants important for swine health, and when zoonotic transmission occurs, public health officials can observe trends in ISU *FLU*ture with the variant IAV strain associated with circulating strains of IAV in swine populations to understand the context of the human health findings.

## Conclusions

ISU *FLU*ture offers a near real-time visualization of genetic changes and epidemiology associated with IAV in swine. The ISU *FLU*ture database is compiled from the 6,186 IAV positive diagnostic cases that were submitted to the ISU VDL to date. Sequence data were then processed and the evolutionary history and spatial and temporal dynamics were assessed. Given the confidentiality of privileged information shared by the client with the ISU VDL, only the USDA subset of the genetic sequence information is available. However, aggregating this confidential data in a secure database and visualizing trends in IAV diversity through space and time by ISU *FLU*ture is a powerful tool to facilitate and complement other tools available for IAV in external sequence databases (GenBank) and analysis sites (IVR and IRD) [29, 30]. The case summary data displayed in the ISU *FLUture* charts and graphs on each webpage is available for download and analysis by investigators. A specific link is also provided on the webpage for downloading unique strain identifiers associated with publicly available HA and NA sequences depicted in the ISU *FLUture* graphs and heat maps. The alphanumeric strain identifiers provided in the download are assigned through the USDA surveillance system and embedded in IAV strain names and can be used to locate the available sequences in GenBank. The ISU *FLU*ture database is updated at daily intervals to give an accurate and current view of IAV activity in influenza from cases submitted to the ISU VDL. These data provide a unique insight into how swine IAV are evolving. They can provide immediate objective criteria to facilitate the rational design of vaccines and state or regional control efforts by veterinarians and pork producers, and the observed genetic diversity may be used to predict antigenic phenotype [44] to describe future virus dynamics. These data have the added advantage of including diagnostic lab data along with sequences available in GenBank in the analyses. Further, these data may be integrated into interspecies transmission investigations of IAV for identification of novel IAV in swine or other hosts.

## Additional files

Additional file 1: **Figure S1.** Reference tree used as a backbone for defining neuraminidase (NA) clades. Two distinct subtypes of NA are found in swine, N1 and N2. Clades of the N1 subtype found in swine include N1.Classical (red), N1.Pandemic (yellow), and N1.Human-Seasonal (orange). Clades of the N2 subtype found in swine include N2.1998 (green), N2.2002 (brown), and the N2.Human-seasonal (blue). The reference tree was built using FastTree2 v2.1.9 [32] to infer the best-known maximum-likelihood tree implementing a general time reversible model of nucleotide substitution with a CAT model of rate heterogeneity with branch lengths rescaled to optimize the Gamma20 likelihood. (PDF 9 kb)

Additional file 2: Nexus format phylogenetic tree. Neuraminidase Reference Sequences. Reference sequences used for determining the clade a neuraminidase sequence falls into. (TXT 16 kb)

Additional file 3: **Figure S2.** Reference tree used as a backbone for defining hemagglutinin (HA) H3 subtype clades. The defined H3 clades are Cluster I (purple), Cluster II (lilac), Cluster III (navy), Cluster IV(red), Cluster IVA (orange), Cluster IVB (mustard), Cluster IVC (light green), Cluster IVD (dark green), Cluster IVE (seafoam), Cluster IVF (blue), human-like (magenta). Human seasonal (black). The reference tree was built using FastTree2 v2.1.9 [32] to infer the best-known maximum-likelihood tree implementing a general time reversible model of nucleotide substitution with a CAT model of rate heterogeneity with branch lengths rescaled to optimize the Gamma20 likelihood. (PDF 7 kb)

Additional file 4: Nexus format phylogenetic tree. Hemagglutinin H3 Subtype Reference Sequences. Reference sequences used for determining the clade an H3 subtype hemagglutinin sequence falls into. (TXT 29 kb)

Additional file 5: **Figure S3.** Reference tree used as a backbone for defining hemagglutinin (HA) H1 subtype clades. The defined H1 clades are H1-α (global nomenclature 1A.1 and 1A.1.1, red); H1-β (1A.2, orange); H1-γ2 (1A.3.2, light green); H1-pdm09 (1A.3.3.2, blue); H1-γ (1A.3.3.3, green); H1-δ1 (1B.2.2, navy); H1-δ1a (1B.2.2.1, dark purple); H1-δ1b (1B.2.2.2, light purple); and H1- δ2 (1B.2.1, magenta). Human seasonal sequences are in black. The reference tree was built using FastTree2 v2.1.9 [32] to infer the best-known maximum-likelihood tree implementing a general time reversible model of nucleotide substitution with a CAT model of rate heterogeneity with branch lengths rescaled to optimize the Gamma20 likelihood. (PDF 5 kb)

Additional file 6: Nexus format phylogenetic tree. Hemagglutinin H1 Subtype Reference Sequences. Reference sequences used for determining the clade an H1 subtype hemagglutinin sequence falls into. (TXT 21 kb)

## Abbreviations

CT: Cycle threshold
HA: Hemagglutinin
IAV: Influenza A virus
LIMS: Laboratory information management system
NA: Neuraminidase
RT-qPCR: Reverse transcriptase quantitative polymerase chain reaction
