# Supplementary figures and images for: ISU FLUture: a veterinary diagnostic laboratory web-based platform to monitor the temporal genetic patterns of Influenza A virus in swine
Source: BMC Bioinformatics. 2018 Nov 1;19:397. doi: 10.1186/s12859-018-2408-7 (PMC6211438; doi:10.1186/s12859-018-2408-7)

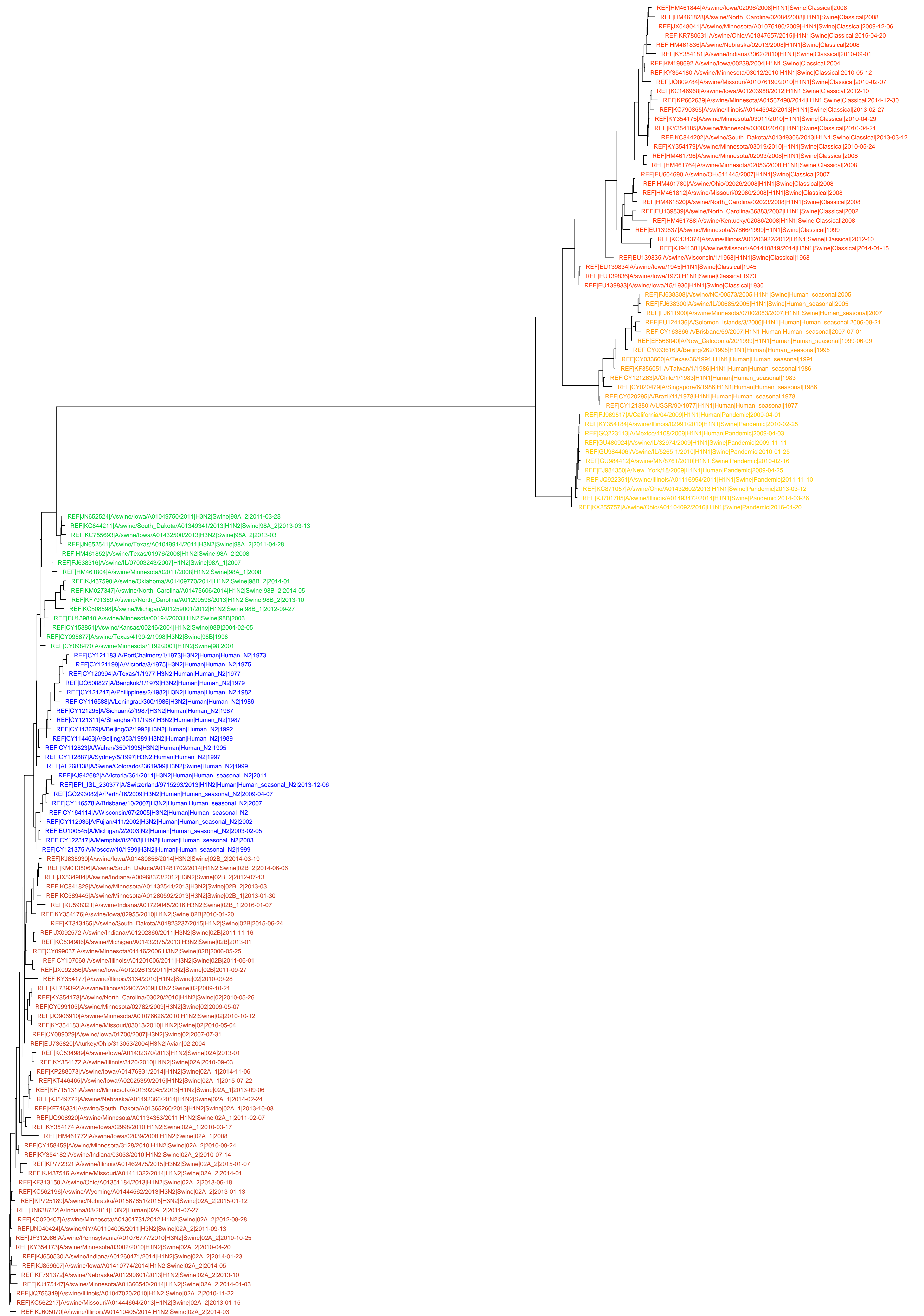

Supplement: Supplementary file 1 — Figure S1. Reference tree used as a backbone for defining neuraminidase (NA) clades. Two distinct subtypes of NA are found in swine, N1 and N2. Clades of the N1 subtype found in swine include N1.Classical (red), N1.Pandemic (yellow), and N1.Human-Seasonal (orange). Clades of the N2 subtype found in swine include N2.1998 (green), N2.2002 (brown), and the N2.Human-seasonal (blue). The reference tree was built using FastTree2 v2.1.9 [32] to infer the best-known maximum-likelihood tree implementing a general time reversible model of nucleotide substitution with a CAT model of rate heterogeneity with branch lengths rescaled to optimize the Gamma20 likelihood. (PDF 9 kb) [file 12859_2018_2408_MOESM1_ESM.pdf]

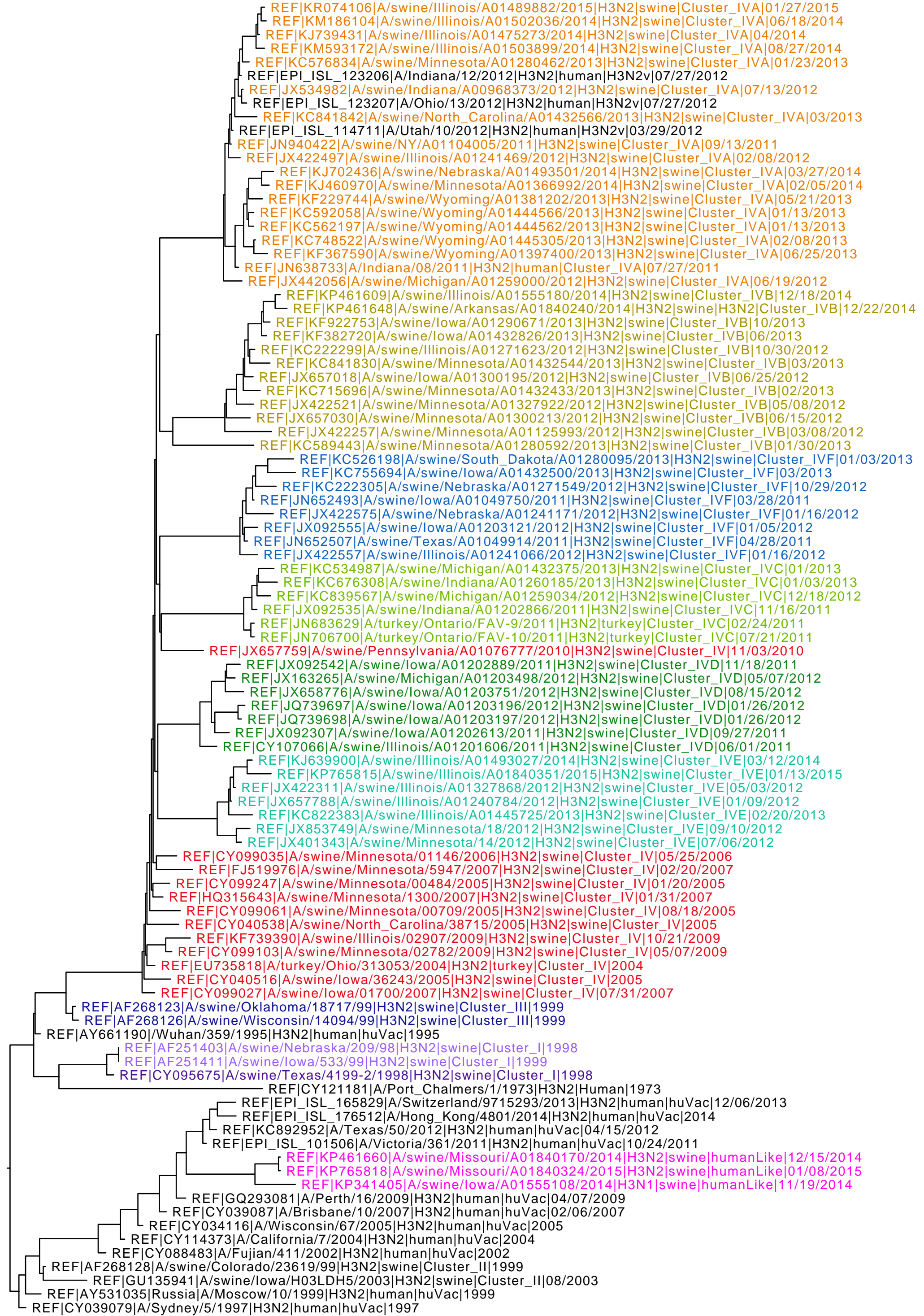

Supplement: Supplementary file 3 — Figure S2. Reference tree used as a backbone for defining hemagglutinin (HA) H3 subtype clades. The defined H3 clades are Cluster I (purple), Cluster II (lilac), Cluster III (navy), Cluster IV(red), Cluster IVA (orange), Cluster IVB (mustard), Cluster IVC (light green), Cluster IVD (dark green), Cluster IVE (seafoam), Cluster IVF (blue), human-like (magenta). Human seasonal (black). The reference tree was built using FastTree2 v2.1.9 [32] to infer the best-known maximum-likelihood tree implementing a general time reversible model of nucleotide substitution with a CAT model of rate heterogeneity with branch lengths rescaled to optimize the Gamma20 likelihood. (PDF 7 kb) [file 12859_2018_2408_MOESM3_ESM.pdf]

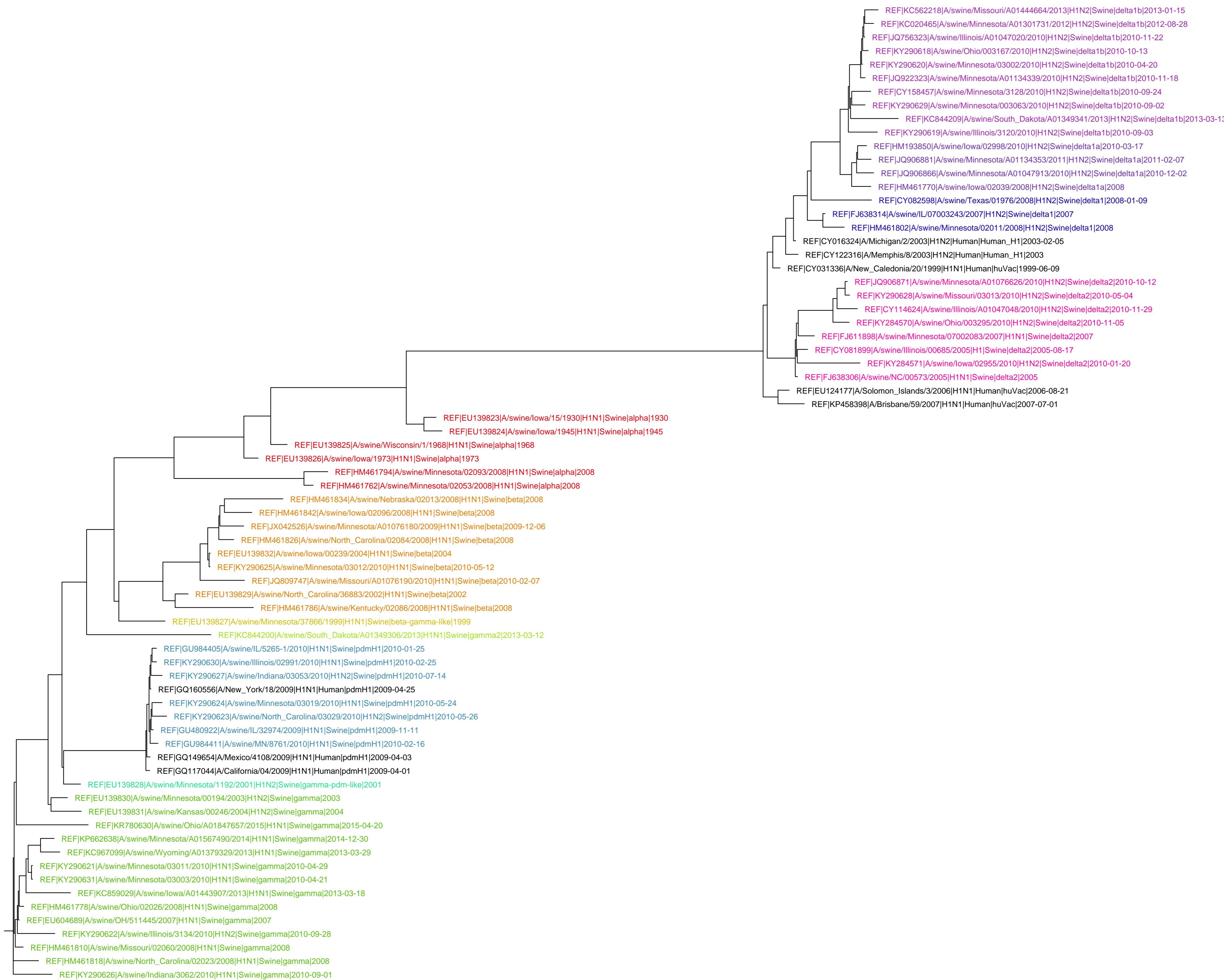

0.06

Supplement: Supplementary file 5 — Figure S3. Reference tree used as a backbone for defining hemagglutinin (HA) H1 subtype clades. The defined H1 clades are H1-α (global nomenclature 1A.1 and 1A.1.1, red); H1-β (1A.2, orange); H1-γ2 (1A.3.2, light green); H1-pdm09 (1A.3.3.2, blue); H1-γ (1A.3.3.3, green); H1-δ1 (1B.2.2, navy); H1-δ1a (1B.2.2.1, dark purple); H1-δ1b (1B.2.2.2, light purple); and H1- δ2 (1B.2.1, magenta). Human seasonal sequences are in black. The reference tree was built using FastTree2 v2.1.9 [32] to infer the best-known maximum-likelihood tree implementing a general time reversible model of nucleotide substitution with a CAT model of rate heterogeneity with branch lengths rescaled to optimize the Gamma20 likelihood. (PDF 5 kb) [file 12859_2018_2408_MOESM5_ESM.pdf]
